# Non-Contact Interaction Between Phorbol Myristate Acetate and Aqueous Alcohol Solutions Under Combined Magnetic Fields

**DOI:** 10.3390/molecules29235814

**Published:** 2024-12-09

**Authors:** Vadim V. Novikov, Elena V. Yablokova, German O. Stepanov, Natalia N. Rodionova, Sergey A. Tarasov, Ekaterina V. Buravleva, Olga I. Yablonskaya, Vladimir L. Voeikov

**Affiliations:** 1 Institute of Cell Biophysics of the Russian Academy of Sciences, 142290 Pushchino, Russia; e.v.yablokova@mail.ru; 2 OOO «NPF «Materia Medica Holding», 129272 Moscow, Russia; stepanovgo@materiamedica.ru (G.O.S.); rodionovann@materiamedica.ru (N.N.R.); satarasovmail@yandex.ru (S.A.T.); 3 Institute of General Pathology and Pathophysiology, 125315 Moscow, Russia; 4 Faculty of Biology, M.V. Lomonosov Moscow State University, 119991 Moscow, Russia; b_u_k_a@mail.ru (E.V.B.); v109028v1@yandex.ru (V.L.V.); 5 N.M. Emanuel Institute of Biochemical Physics RAS, 119334 Moscow, Russia

**Keywords:** weak magnetic field, combined magnetic field, high dilutions, non-contact interactions, water, neutrophils, chemiluminescence, ROS

## Abstract

Previous research has demonstrated that a combined magnetic field (CMF) plays a critical role in modifying the properties of aqueous solutions, leading to an increase in the luminol-enhanced chemiluminescence of neutrophils. Using this model, the distant interaction between aqueous solutions was demonstrated, and the role of a CMF in the regulation of this phenomenon was established. In the current study, highly diluted (HD) phorbol myristate acetate (PMA) solution (the donor) was incubated with aqueous ethanol (the acceptor), both in a CMF-generating device and under geomagnetic field (GMF), for 0, 20, and 60 min. After a 60 min incubation at a 0 cm distance with HD PMA under both GMF and CMF, acceptor samples added to neutrophils increased neutrophil chemiluminescence by approximately sevenfold. The ability of HD PMA, which had been incubated with an acceptor, to activate ROS production diminished within 60 min of observation. However, the HD PMA sample remained an effective donor for up to 6 days after preparation. At a 10 cm distance between the donor and acceptor, the activation of the acceptor did not occur. These findings provide new insights into the phenomenon of distant interaction of solutions, whose mechanisms are suggested to be related to the quantum electrodynamics of water molecular dynamic structures.

## 1. Introduction

Over the past decades, various authors have reported that weak magnetic fields (WMFs) can affect the ionic strength, pH, surface tension, and viscosity of aqueous solutions [1,2]. In the literature, one can find evidence that exposure to WMFs enhances the internal vibrations of water molecules, thereby increasing their polarizing effect and their movement ability. As a result, water uptake into cells is enhanced [3]. Magnetic fields were reported to change hydrogen bond strength [4] while they weaken the van der Waals interactions between water molecules, thereby contributing to hydrogen bond strengthening [5] and water cluster growth [6]. Magnetic fields are shown to be able to increase the number of hydrogen bonds in water, increasing cohesion and decreasing water flow through carbon nanotubes [7].

According to other authors, physicochemical effects on water and ice after treatment with WMFs are associated with different proton structural states. It has been suggested that WMF influences the likelihood of proton transfer among clusters of water molecules, with a varying impact depending on the presence of impurities in the water. The lasting memory effects of very weak magnetic fields over extended periods (5–6 h) were reported [8].

Meanwhile, in the absence of a magnetic field, biological processes cannot occur normally. It was shown that under zero magnetic field conditions, *D. melanogaster* development was disturbed, supposedly because of changes in membrane proteins and genes involved in ion flow transport through the membranes of neural cells [9]. This supports the idea that WMFs are an important part of the natural environment that has not changed significantly during a very long period of life development on Earth and act as moderators of processes in living organisms, while the latter can also emit electric and magnetic fields.

Previously, we have shown that water treated with WMFs possesses altered biological properties [10,11,12]. Measurements of luminol-enhanced chemiluminescence (CL) following the respiratory burst of neutrophils have been used to demonstrate these biophysical effects on water and aqueous solutions treated with WMFs [13].

A neutrophil respiratory burst is a rapid release of reactive oxygen species (ROS) from neutrophils that destroy invading pathogens [14]. This process is often studied using phorbol 12-myristate 13-acetate (PMA), an activator of protein kinase C, which induces oxygen consumption, followed by a strong respiratory burst in neutrophils [15]. Luminol acts as a chemiluminescent probe for hydrogen peroxide and superoxide anions. The emitted light upon reaction with ROS can be detected and quantified, providing a sensitive measure of ROS production.

Earlier, we demonstrated that introducing a water sample pre-treated with a combined magnetic field (CMF) (60 μT static magnetic field (SMF) and a 100 nT alternating magnetic field (AMF) with a frequency of 12.6 Hz) to a suspension of neutrophils resulted in an approximate 70% increase in ROS generation [16]. This ability to stimulate ROS production persisted after CMF-pretreated water was subjected to the serial dilution procedure with vigorous shaking. However, when the dilution process was conducted in a specialized chamber under zero magnetic field conditions, the enhancement in ROS production was not observed. Aqueous solutions (including high dilutions (HDs)) of several proteins and low molecular weight regulators of neutrophil activity were also pre-treated with CMF, which resulted in their altered effects compared to non-pretreated controls.

There was an important observation that if CMF-pretreated water and neutrophil suspension samples were kept in close proximity, this often led to unexpected CL measurements, suggesting a distant interaction between them. Further experiments demonstrated that incubation in close proximity of HD antibodies to interferon-gamma (IFNγ) and water or a solution of IFNγ under CMF conditions led to a significant increase in ROS generation when water or IFNγ solution was subsequently added to neutrophils (185–356% increase, *p* < 0.05 compared to control) [13]. This increase was not observed under zero magnetic field conditions. Therefore, we suggested that a sample of HD antibodies to IFNγ was a donor (the source of the physical stimulus/signal) and water or IFNγ solution were acceptors (that absorb the stimulus/signal from the donor without direct contact with the liquids).

We previously discovered the ability of an HD substance to influence substances in adjacent vials [13]. A similar phenomenon has been reported by other authors [17,18,19,20,21,22,23,24,25]. Infrared emission spectra of IFNγ surrounded by HD solutions of IFNγ or antibodies to IFNγ differed from the emission spectra of IFNγ surrounded by control water [18], and a change in the IFNγ hydration shell was observed [17]. The ability of donors to influence the physicochemical parameters (pH, conductivity, and redox potential) and UV/Vis spectra of acceptors was also detected [20]. The theoretical results obtained by [21] make it possible to interpret the distant interaction of supramolecular systems of aqueous solutions not only as a physicochemical process but also as an informational process. Experimentally, it was shown that DNA molecules can transduce information through the emission of particular electromagnetic signals [22].

At the moment, there is a lack of research that could further describe and explain the phenomenon of non-contact interactions. The main objective of this study is to investigate the distant interaction of HD PMA as a donor and 0.5% aqueous ethanol solution as an acceptor. We also aim to obtain evidence on the role of magnitude and frequency of CMF in this process. Using the respiratory burst of neutrophils as a model, we will assess the importance of the distance between “donor” and “acceptor” solutions during joint incubation. We also intend to investigate the relaxation of the donor (HD PMA), which is the loss of its ability to affect the acceptor (0.5% ethanol solution) during 7-day-long storage. We will also study if the donor preserves its properties to affect the acceptor for 1 h after it has been jointly incubated with the acceptor; in other words, we will test whether it can be used as a donor more than once.

## 2. Results

### 2.1. Effect of Duration of HD PMA Joint Incubation with Acceptor Solution Under CMF Conditions on Neutrophil CL Intensity

In continuation of our research into the possibility of distant interaction between HD solutions of biologically active substances and 0.5% ethanol solution in highly purified water in donor–acceptor pairs under CMF conditions [12,13,16], we conducted a series of experiments with HD PMA. We studied the possible influence of the time of the joint incubation of HD PMA samples and acceptor samples on the acceptor’s ability to change neutrophil activity in a manner that PMA could achieve.

**Figure 1:**
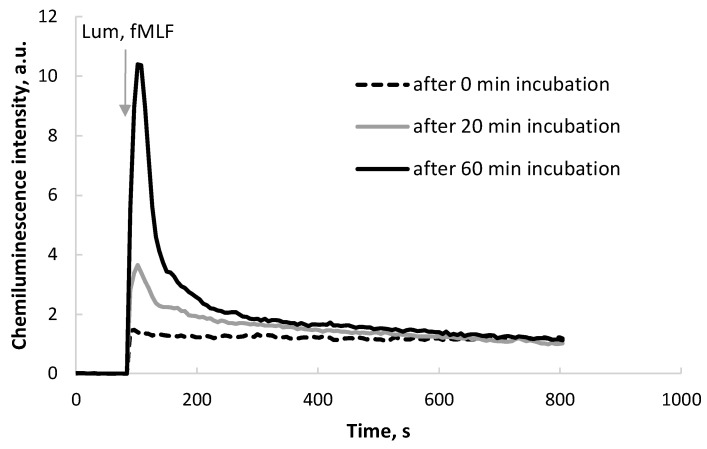
Representative CL kinetic curves of a neutrophil suspension after the addition of 0.5% aqueous ethanol acceptor aliquot preincubated with HD PMA (donor) in CMF (a combination of SMF of 60 µT and AMF of 100 nT at a frequency of 48.5 Hz) for various time periods.

Figure 1 represents typical kinetic curves obtained by the induction of ROS generation by neutrophils. After respiratory burst stimulator fMLF and luminol were added to the cells, a CL increase was recorded, which was 100 to 1000 times higher than the background CL values of neutrophils.

Some prepared acceptor samples have not been in contact with the donor cuvettes (HD PMA). They were kept under GMF conditions and were used as controls. As shown in Figure 1, there is a noticeable increase in the CL response after the interaction of neutrophils with acceptor samples preincubated with HD PMA for 20 or 60 min compared to the “0 min” sample. In this case, the longer incubation time (60 min) is obviously associated with the maximum peak height of the CL kinetic curve.

**Figure 2:**
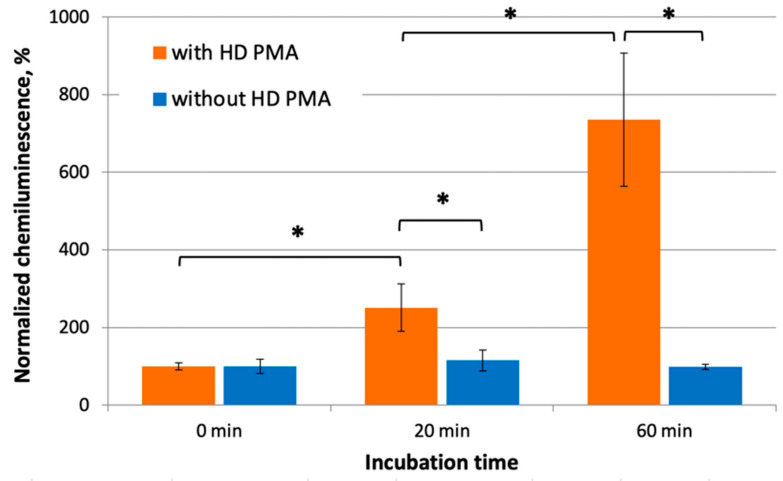
Normalized maximum CL intensity of a neutrophil suspension after the addition of 0.5% aqueous ethanol (acceptor) preincubated with HD PMA (donor) samples (orange bars) or separately (blue bars) in CMF (a combination of SMF of 60 µT and AMF of 100 nT at a frequency of 48.5 Hz) for various periods of time. “0 min incubation” stands for CL values for the control acceptors before incubation with donor samples. Bars reflect the maximum CL intensity after normalization using the “0 min incubation” sample. Data are presented as arithmetic means ± SD, n = 6. *—*p* < 0.05 (in comparison with “0 min” using Wetch’s *t*-test).

CL measurements of a series of similar experiments are presented in Figure 2. The incubation of 0.5% aqueous ethanol samples jointly with samples containing HD PMA (Figure 2, orange bars) for 20 and 60 min resulted in an increase in ROS production by neutrophils by an average of 2.5 and 7 times, respectively, compared to control measurements made before joint incubation. Data from control measurements before joint incubation were taken as 100%, and normalization was carried out relative to mean values for control samples. Normalized maximum CL values in the group of samples after 60 min of incubation differ significantly both from the group of samples after 0 min of incubation and from the samples that were incubated for only 20 min (*p* < 0.05). Bartlett’s test revealed that the data were not homogenous, so Welch’s t-test was used for statistical processing. This proved that normalized maximum CL values after 20 and 60 min of the joint incubation of the donor and acceptor were significantly higher compared to the “0 min” control (*p* < 0.05) and different from each other.

In a “sham” experiment conducted under the same conditions (Figure 2, blue bars), but in the absence of donor samples, the normalized maximum CL values of these samples were not statistically significantly different from CL values of the “0 min” samples and did not depend on the incubation time. Thus, the ability of the donor (HD PMA) to have a distant effect on samples of the acceptor (0.5% aqueous ethanol) was demonstrated.

### 2.2. Effect of Duration of HD PMA Joint Incubation with Acceptor Solution Under GMF Conditions on Neutrophil CL Intensity

**Figure 3:**
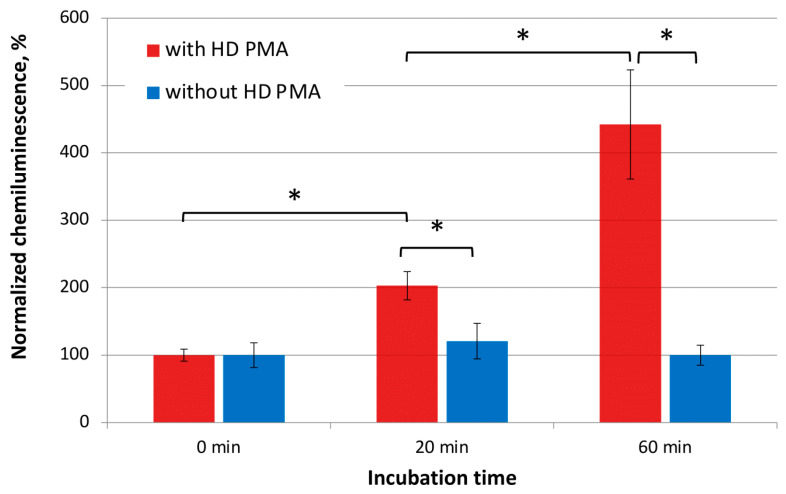
Normalized maximum CL intensity of a neutrophil suspension after the addition of 0.5% aqueous ethanol (acceptor) preincubated with HD PMA (donor) samples (red bars) or separately (blue bars) in GMF for various periods of time. A “0 min incubation” stands for CL values for the control acceptors before incubation with donor samples. Bars reflect the maximum CL intensity after normalization using the “0 min incubation” sample. Data are presented as arithmetic means ± SD, n = 6. *—*p* < 0.05 using Welch’s *t*-test.

Since the first series of experiments was carried out under conditions of CMF with certain specified parameters, it was of interest to find out whether a similar effect of the donor samples containing HD PMA on the acceptor may occur in GMF conditions. For this purpose, the next series of experiments was carried out using GMF. The results of these experiments are presented in Figure 3.

Just as under conditions of a CMF with a variable frequency of 48.5 Hz and shielding from external GMF, under conditions of a natural geomagnetic background, the effect of joint incubation of the donor (HD PMA) and acceptor (0.5% aqueous ethanol) for 20 and 60 min on ROS generation by neutrophils was still present, although it was somewhat less pronounced (Figure 3, red bars). After 20 min of joint incubation in close contact, the acceptor (0.5% aqueous ethanol) samples caused a two times stronger neutrophil activation than the control samples, where the acceptors were incubated without the donor. After 60 min, the normalized maximum CL values became 4.4 times higher than in the “0-min incubation” control. Similar to experiments with CMF (Figure 2), statistically significant differences were observed between acceptor samples that were incubated for 20 and 60 min (*p* < 0.05). According to Bartlett’s test, the data were not homogenous, so Welch’s t-test was used for statistical analysis.

In a “sham” experiment conducted under the same conditions, but in the absence of donor samples, no significant differences were observed between groups of 0.5% aqueous ethanol samples, which were incubated in GMF for various periods of time in the absence of HD PMA samples (Figure 3, blue bars).

At the next stage of the work, it was necessary to find out whether the ability of HD PMA preparations to affect the acceptor changes over time.

### 2.3. Change in HD PMA’s Ability to Act as a Donor After Repeated Incubations Over a Week

Studying the ability of HD PMA (donor) to have an effect on the acceptor (freshly prepared 0.5% aqueous ethanol solution) was the purpose of the following experiment. A CMF with an AMF component oscillating at a frequency of 12.6 Hz was used for joint incubation.

**Figure 4:**
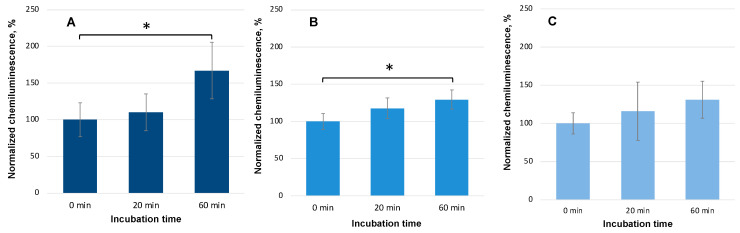
Normalized maximum CL intensity of a neutrophil suspension after the addition of the acceptor (0.5% aqueous ethanol) incubated together with the donor (HD PMA) in CMF (a combination of SMF of 60 µT and AMF of 100 nT at a frequency of 12.6 Hz) for various periods of time. A “0 min incubation” stands for CL values for the control acceptors before incubation with donor samples. Bars reflect the maximum CL intensity after normalization using the “0 min incubation” sample. Data are presented as arithmetic means ± SD, n = 6. An aliquot of donor for co-incubation was taken (**A**) on the day of preparation of HD PMA; (**B**) 6 days after preparation of HD PMA; and (**C**) 7 days after preparation of HD PMA. *– statistically significant differences, according to Student’s *t*-test (*p* < 0.05).

Acceptor samples incubated with HD PMA on the first day after preparation caused an increase in the chemiluminescent response of neutrophils (see Figure 4A). Statistically significant differences were recorded after 60 min of the joint incubation of the donor and acceptor in the controlled magnetic field-generating device. Neutrophil activation was 1.67 times higher than in the control (“0 min of incubation”). Acceptor samples incubated with the donor for 20 min only showed a tendency to stimulate the respiratory burst.

After 6 days of storage, the ability of HD PMA to affect acceptor samples decreased. The CL of the neutrophils with an addition of the acceptor, which was jointly incubated for 60 min with a donor prepared 6 days earlier, was 1.29 times lower compared to the CL in the neutrophils with the acceptors that were incubated for 60 min with a freshly prepared donor (*p* < 0.05) (see Figure 4B).

On the seventh day after the preparation of HD PMA, a similar experiment revealed a tendency toward the activation of neutrophils (131% compared to the control without joint incubation, *p* > 0.05) after a 60 min joint incubation of the acceptor with the donor (see Figure 4C). The same trend was observed with the samples that were incubated for 20 min: the normalized maximum CL intensity increased to 118% and 116% on the sixth and seventh days, respectively. Thus, a relaxation in time of the ability of HD PMA to affect the acceptor was demonstrated.

### 2.4. HD PMA Relaxation After Interaction with Acceptor

The phenomenon of the relaxation of the donor’s ability to stimulate the activity of 0.5% aqueous ethanol acceptor solution that we have identified led to another series of experiments. Here, we investigated the change in the donor’s ability to activate the neutrophil suspension in a manner PMA did. For this, aliquots of donor samples, which had already been incubated for 20 and 60 min with the acceptor, were added directly to the neutrophil suspensions instead of the acceptor solution, as in all previous experiments.

**Figure 5:**
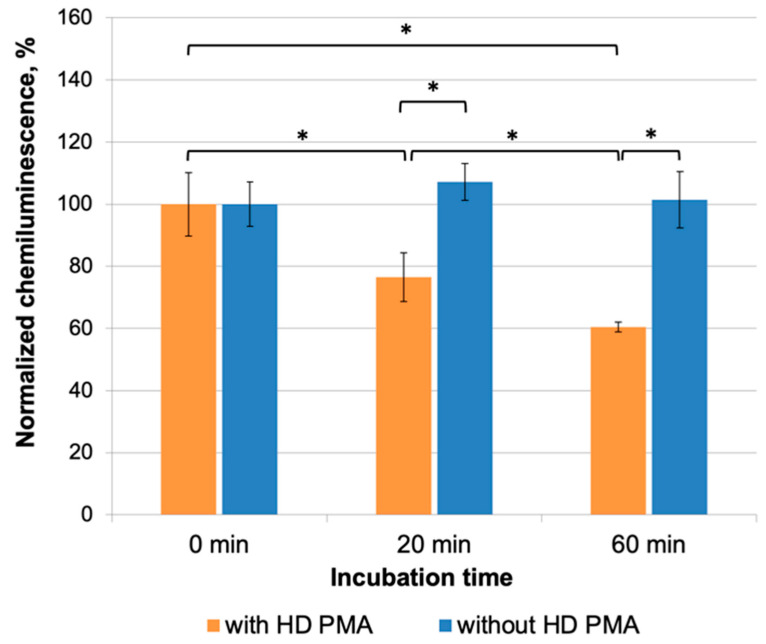
Normalized maximum CL intensity of a neutrophil suspension after the addition of the donor (HD PMA) that had been jointly incubated with the acceptor in close contact (orange bars) or at 10 cm distance (blue bars) for 20 and 60 min. CMF parameters were as follows: SMF at 60 µT and AMF at 100 nT, oscillating in a sinusoidal regime with a frequency of 12.6 Hz. A “0 min incubation” stands for CL values for the control acceptors before incubation with donor samples. Bars reflect the maximum CL intensity after normalization using the “0 min incubation” sample. Data are presented as arithmetic means ± SD, n = 6. *—*p* < 0.05, according to Welch’s *t*-test.

The results are presented in Figure 5 as orange bars. The “0 min” values, taken as 100%, indicate the peak CL intensity without the joint incubation of donors and acceptors under GMF conditions. The orange bars labeled “20 min” and “60 min” indicate the CL intensity when we used donors that had been incubated jointly with the acceptor under CMF conditions (a combination of SMF at 60 µT and AMF at 100 nT, oscillating in a sinusoidal regime with a frequency of 12.6 Hz) in close contact for 20 or 60 min. These donor solutions were less effective in priming neutrophils than the “0 min” sample, and their activity decreased approximately by 20 and 40%, respectively.

The blue bars in Figure 5 represent the data from the “sham control” experiment, in which the donors were incubated with acceptors at a 10 cm distance in the CMF. As can be seen from the figure, there is no significant difference between the CL values obtained from donor samples that were incubated under CMF conditions for 20 min, 60 min, or 0 min (used in the experiment immediately after preparation). These results demonstrate that the decrease in CL intensity is likely associated with joint incubation when the donor and acceptor tubes are placed in close proximity. At a distance of 10 cm, there was no evidence of interaction between donors and acceptors.

## 3. Discussion

The presented research develops the topic of distant interactions between substances that are placed in different containers. In particular, one of the substances (PMA) acted as a donor of a certain physicochemical signal and was used in an extremely high dilution, while another, 0.5% aqueous ethanol, acted as an acceptor of this signal. Afterward, this acceptor solution’s ability to “absorb” the properties of PMA in a chemiluminescent assay with neutrophil respiratory burst was examined. As we showed previously [12,13,16], the magnetic field plays a crucial role in the ability to transfer the signal. In this study, samples were under the conditions of GMF and CMF, with specific parameters of constant and alternating components that were also determined in earlier research. In this study, we focused on determining the effect of the time of joint incubation on the signal transmittance from the donor to the acceptor in different magnetic conditions, as well as a relaxation of the donor solution and changes in its ability to act as the signal donor over a period of 7 days.

We show that a 20 min joint incubation of the donor and the acceptor in the CMF (a combination of SMF of 60 µT and AMF of 100 nT at a frequency of 48.5 Hz) in the controlled magnetic field-generating device leads to a significant increase in the ability of the acceptor to activate neutrophils. This resulted in elevated luminol-enhanced CL. Interestingly, the acceptor samples that were outside the controlled magnetic field-generating device in GMF conditions with the donor 200 cm away did not have such an effect (Figure 2). Longer incubation (60 min) of the donor with the acceptor led to a more pronounced effect when CL increased by nearly seven times. These results indicate that there is a phenomenon of distant interaction that depends on the exposure time. It is of interest to carry out sessions with even longer co-incubation. The results of a “sham” control experiment with only the acceptor exposed to the CMF prove that this effect is not due to the CMF treatment itself.

The same trend, however, slightly less pronounced, was demonstrated when the donor and acceptor samples were placed outside the device in the conditions of GMF in close proximity to each other for 20 and 60 min (Figure 3). This proves that such interaction does not depend on artificial laboratory conditions and occurs normally in nature.

The following question may arise: for how long after preparation does the HD donor possess its ability to affect the acceptor? It was shown that on the sixth day after preparation, the donor (HD PMA) could activate the acceptor in the presence of the CMF (a combination of SMF (60 μT) and AMF (100 nT), which was formed according to the sinusoidal regime at a frequency of 12.6 Hz). This resulted in enhanced CL in neutrophils (Figure 4). On the seventh day of storage, the donor’s ability to activate the acceptor was trend-like and not significant. This finding shows that the state of the donor is also important for efficient signal transfer. To find out more about changes in the donor in the course of signal transduction, we carried out another experiment that gave some interesting results.

We found that the donor itself loses its “power” when it transduces the physicochemical signal to the acceptor in CMF conditions (Figure 5). This type of discharge is more pronounced after longer joint incubation. The close proximity of the donor and acceptor is of major importance, as even a 10 cm distance is too big for the substances to interact.

Thus, we have made a few steps forward in describing and understanding the phenomenon of distant interactions of substances. In particular, not only the magnetic field parameters matter but also the exposure duration, freshness of the donor sample, and the distance between donor and acceptor.

One may ask why HD PMA was used as a donor rather than a sample with conventional concentration. The possible answer lies in paradoxical properties of HDs of substances. From the perspective of conventional chemistry and biochemistry, the activity of substances in a solution should decrease in proportion to the level of dilution. Nonetheless, studies show that HDs below and above Avogadro’s number possess unexpected effects given that the dilution process included specific physical treatments, such as mechanical shaking, rotation, and exposure to electromagnetic fields [18]. Other authors [19,20] suggested calling such solutions processed liquids that held molecular imprints of biologically active compounds that were originally present in conventional physiological concentrations. These physical treatments make HD solutions the sources of molecular information that can be transferred to other substances and living organisms [18,23,24].

Luc Montagnier, a Nobel laureate, directed research [25] in which he and his co-authors found that filtrates from microorganisms, DNA, and their highly diluted solutions emitted an extremely low frequency (ELF) electromagnetic field (500−3000 Hz). Interestingly, the plasma of humans infected by these microorganisms also had these signals.

In an experiment, they obtained an HIV 487 base pair-long DNA fragment, multiplied it with PCR, diluted it by 10-fold several times, and detected a specific ELF signal from some of the dilutions. Then, they filtered one of the emitting diluted DNA samples, put it in a container made of a shielding alloy, together with a filtered pure water sample, and generated a magnetic field with a copper solenoid that received a low-intensity electric current oscillating at 7 Hz. After an 18 h joint incubation in the presence of the magnetic field, the pure water sample and its dilutions started to emit the same signal as the diluted DNA fragment sample.

The mechanism of such signal emergence and its action is unclear, and most speculations rely on observations only. Obviously, the nature of signal transfer from HD donor to acceptor is physical. The mechanical vibration (shaking) is believed to be the key component of turning an HD sample into a donor that can distantly affect the acceptor, not the highly diluted substance itself. Research [26] shows that in jointly incubated closed vials (one contained a substance (for instance, a solution of antibodies to IFNγ), and another contained water), vibrational treatment resulted in changing physicochemical properties of water (pH, conductivity, the ability to emit in the radio range, and biochemical activity). It is quite possible that the distant interactions between such water samples and the distant interactions of HD PMA shown in our study are of a similar nature.

However, the most comprehensive approach to understanding the mechanism of action of HD solutions is reflected in the concept of coherent domains (CDs) in water proposed by Del Giudice and Preparata and grounded in quantum electrodynamics (QED) [27]. It challenges classical interpretations of water’s properties by explaining certain anomalies in water’s behavior, such as its unique thermal and dielectric properties, through the lens of quantum physics.

Coherence represents the macroscopic expression of the microscopic dynamics of elementary parts, when specific boundary conditions are fulfilled, such as maintaining a density above a critical threshold and a system temperature below a critical value [28]. In an ensemble of quantum particles, fluctuations between internal states cause the emission or absorption of EMF. When particle density exceeds a critical threshold and temperature is below a certain limit, the system transition to a coherent state occurs. In this state, the particles and EMF couple, oscillating in phase. The EMF is trapped within a coherent system to prevent outward radiation. This coupled oscillation defines the system’s lowest energy state, the quantum vacuum.

For liquid water, the energy of an oscillating molecule at room temperature is 1.53 eV, with the average energy of the induced EMF of 3.55 eV per molecule. The attractive interaction energy between the field and molecule is −5.34 eV, giving a net balance of −0.26 eV in the case of coherence, compared to the non-coherent particle case [29]. Coherent oscillation leads to a lower energy state for the whole system, making the formation of CDs energetically beneficial, which stabilizes the domain [30,31].

When exposed to the EMF, the electronic states of water molecules can become excited and shift to higher energy states. This excitation causes the molecules to oscillate with a certain frequency. When the abovementioned critical conditions are reached, the excited electronic states of water molecules start to oscillate in phase, reaching the minimum energy of a system, and this is the condition for coherence emerging. This phase synchronization extends throughout the region, forming a 100 nm CD, in which millions of molecules oscillate coherently. A CD attracts other water molecules from the environment that are able to resonate with the growing EMF [29]. The chemical and biological properties of water that is rich with coherent phases are different from those of non-coherent water in many ways [32].

There are arguments that Brownian motion and thermal fluctuations could, in theory, disrupt any ordered structure in water. However, quantum coherence within the CD implies that the behavior of each molecule is not independent but is correlated with the entire domain. This collective behavior creates a robust structure that is less susceptible to random disturbances from individual molecular motions. Moreover, the energy gap between the CD and the environment acts as a barrier that insulates the CD from the surrounding thermal noise. To disrupt the coherent state, sufficient energy is required to overcome this gap, which is typically much higher than the energy provided by thermal fluctuations. CDs can be viewed as dissipative structures that maintain their order by dissipating energy to the surroundings. This continuous energy exchange helps sustain the coherent state against disruptive forces and possibly can be transferred to the surrounding objects.

It was suggested that the HD preparation process is crucial for the formation of coherent domains (CDs) in water, and that the specific water clusters of water molecules are rather stable, forming what is called a mesoscopic water phase [31]. CDs store energy in the form of coherent vortices. CDs may lose and obtain new water molecules because thermal oscillations counterbalance the stability of the coherent phase. It is believed that HD signals can be stored and passed on by coherent domains in water [33]. It has been proposed that there are two types of CDs: CD_rot_ and CD_elec_. CD_elec_ contain weakly bound quasi-free electrons, whose magnetic dipoles are aligned with the external magnetic field [34]. CD_rot_ consist of water molecules that coherently oscillate between two of their rotational states. Information can be stored in a complex of several CD_rot_. They possess ferroelectric properties, so information can be preserved in a way comparable to that in materials composed of domains with magnetic moments.

Dr. Yinnon has made significant contributions to understanding how mechanical actions, such as shaking, can alter the properties of water and highly diluted solutions [35]. Mechanical shaking injects energy into the system, enhancing the alignment of water molecules’ dipoles, which is characteristic of CDs. This additional energy helps overcome the natural thermal agitation that disrupts coherence, leading to more extensive and stable coherent domains. According to Yinnon [36], shaking excites or breaks CD_rots_ that coherently oscillate between two of their rotational states. Being added to a new portion of pure water the excited or broken CD_rots_ regenerate new CDs there.

Mechanical agitation induces nanoscale gas bubble generation [37]. The concentration and sizes of these nanobubbles were analyzed using nanoparticle tracking analysis. The authors suggested that the gas–liquid interface played a crucial role in nanobubble formation during the vibrational process. It has been shown that negatively charged nanobubbles might affect the bioelectric field of plants [38]. In another study [39], after vigorous shaking, the levels of molecular oxygen and carbon dioxide in water decreased, and hydrogen peroxide and hydroxyl radicals were produced, increasing with vibration frequency. ROS generation occurrence was possibly associated with the conversion of molecular oxygen from the triplet to the singlet state. This phenomenon can be one of the explanations for increased CL by neutrophils in our experiments. However, if shaking was the only reason for increased CL in the samples, then in all the control samples there also would have been this increase. The control acceptor samples were shaken as well.

Shaking-induced bubble formation and its action are not the same in water and water–alcohol mixtures due to differences in size, charge, and monopole–dipole or positive–negative interactions [40]. The shaking process can help trap gases within these bubbles, preventing their immediate collapse and creating surfaces inside the water medium.

In an AMF, the movement of ions occurs along a cycloidal trajectory in a plane perpendicular to it and with a certain circular (cyclotron) frequency, the value of which depends on the charge, the mass of the ion, and the strength of the magnetic field [41]. This phenomenon is known as ion cyclotron resonance (ICR). The two frequencies of the variable component of CMF that were used in this study were 12.6 Hz (equivalent to the ICR frequency of (H_3_O^+^ (H_2_O)_3_), the so-called Eigen cation) and 48.5 Hz (equivalent to the ICR frequency of H_3_O^+^). Naturally occurring ICR is assumed to play a role in the regulation of living systems, and it can be used purposely to affect the processes in organisms [42,43]. Earlier, it was shown that the excitation of ions with a magnetic field with a frequency that matches their ICR causes the phenomenon of ion ejection [44] and affects ion movements in solutions [45]. Possibly, there is an important role of ICR in CD formation and sustainability in biological objects [46,47,48,49]. Currently, the Liboff-Zhadin effect supported by the ICR theory is the best proof of the QED coherent domain theory.

In summary, our study is devoted to the specifics of non-contact interactions between the HD solutions of biologically active substances and other aqueous solutions. Emphasis is placed on the intricacies of proper preparation techniques for all samples. Our findings accentuate the ability of HD solutions, not only to transfer physicochemical signals through direct contact but also to influence biological systems distantly in the presence of CMF. This opens up a spectrum of possibilities for understanding the distant interactions between substances.

Future research should aim to extend the list of substances that can exert significant biological effects even at high dilutions. A multidisciplinary approach, including physics, chemistry, and biology, is essential for understanding the underlying mechanisms of the signals emitted by HD substances. Such investigations could contribute to therapeutic practices and material science, suggesting new developments in biosciences.

## 4. Materials and Methods

### 4.1. Generating a Controlled Magnetic Field

**Figure 6:**
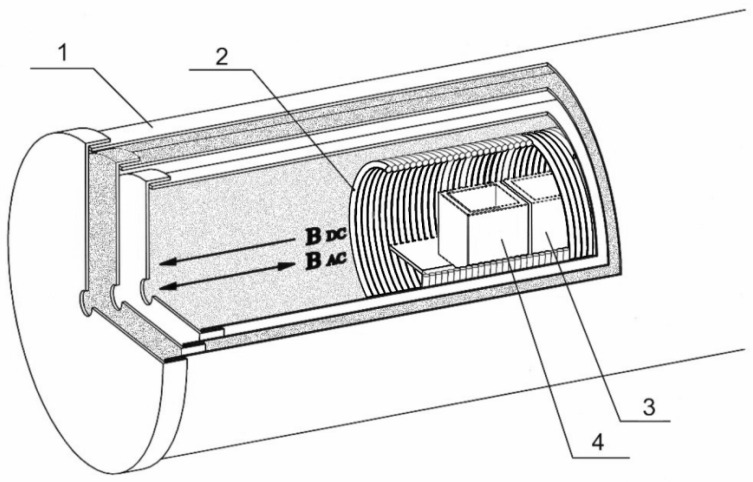
Controlled magnetic field-generating device. 1—Permalloy shields; 2—Solenoid (magnetic copper wire coil); 3—Cuvette with an acceptor sample (aqueous alcohol solution); 4—Cuvette with a donor sample (HD PMA); BAC—Magnetic induction produced by alternating current; BDC—Magnetic induction produced by direct current.

The constant part of the GMF typically ranges from 30 to 65 μT, while the background variable values are determined by the presence of reinforced concrete structures and the parameters of the laboratory’s electrical network (~50 Hz, ~50 nT). To standardize experimental conditions and reduce environmental influence, specialized research equipment was employed: a device designed to generate a controlled magnetic field (Figure 6).

Three 1 mm-thick permalloy cylinders placed coaxially comprised a magnetically shielded chamber, with three-layer permalloy caps on both ends. At one end, there was a perforation for the cable input. The inner space of the chamber was 42 cm long and 22 cm wide. This screening chamber reduced the GMF by ~10,000 times, with the remaining magnetic field being less than 10–20 nT inside and several nT of environmental electromagnetic noise. A Mag-03MS100 fluxgate magnetometer (Bartington Instruments, Witney, UK) was used for magnetic field measurements.

A CMF was induced by applying a current through a copper solenoid with a diameter of 18 cm made of a 1 mm wire. It was 36 cm long and had 720 turns, with a winding resistance of 7.5 Ohm. The walls of the coil were equidistant from the cuvettes with samples placed inside.

A DC power supply was used to induce a static magnetic field (SMF). A digital and analog transducer (DAT) equipped with an L-791 card (L-Card Company, Moscow, Russia) was used to induce an alternating magnetic field (AMF) of a certain frequency and amplitude. The frequency that modulated the variable part of the CMF was equal to the ion cyclotron resonance (ICR) frequency of either the hydrated hydronium ion (12.6 Hz) or the hydronium ion (48 Hz) [48]. The calculation of these frequencies was described previously [11].

### 4.2. Sample Preparation

#### 4.2.1. High Dilutions of PMA (HD PMA)

All solutions were prepared using highly purified water (resistivity of >18.0 MΩ× cm) produced by a Milli-Q Integral 5 water system (Merck, Darmstadt, Germany). Water was stored in 1 L glass bottles with tight caps at room temperature in dim light.

All manipulations were carried out under sterile conditions in dim light at room temperature. The stock solution of PMA (20 nM PMA (Sigma-Aldrich, Saint Louis, MO, USA)) was used in the experiments on the same day it was prepared. HD PMA was obtained through serial dilutions of the stock solution in a water–ethanol mixture (20% *v*/*v*). The final dilution was made using pure water, to which ethanol was added to obtain a 0.5% solution. The procedure was performed under GMF conditions using a method similar to the one described previously [12]. Briefly, the first dilution was 10-fold, and all subsequent dilutions were at a 1:100 ratio. In total, there were 50 dilutions, with vigorous shaking between them performed by hand at a frequency of ~4 Hz (21 strokes in 4.8 s). The volume of each dilution was 5 mL. The final solution was kept at room temperature. The theoretical level of reduction in the concentration of PMA was at least 10^24^ times.

#### 4.2.2. Neutrophil Suspension Isolation

This study was performed using peritoneal-induced neutrophils from specific pathogen-free outbred CD-1 line mice. The mice were obtained from the nursery of laboratory animals at the M.M. Shemyakin and Yu.A. Ovchinnikov Institute of Bioorganic Chemistry, Russian Academy of Sciences. The average weight of the experimental animals was 25 ± 1 g; the mice were 4 months old.

Each mouse received a single intraperitoneal injection of 150 μL of a suspension of opsonized zymosan from *Saccharomyces cerevisiae* at a concentration of 5 mg/mL (Sigma-Aldrich, St. Louis, MO, USA). Fifteen hours later, the mice were euthanized via cervical dislocation, and neutrophils were isolated, as described previously [11]. Briefly, the suspension of peritoneal cells was obtained by washing the peritoneal cavities with 4 mL of cold calcium-free Hank’s solution. The cell suspension was pipetted and centrifuged at 600× *g* for 5 min. The supernatant was removed, and the neutrophil-enriched pellet was resuspended in 4 mL of cold calcium-free Hank’s solution. The mixture was then left for 60 min at 4 °C.

The cell number was determined using a hemocytometer. A trypan blue exclusion test was used to determine the number of viable cells present in the suspension. For use in CL analysis, a suspension of neutrophils (≥98% viable) was diluted with modified Hank’s medium (138 mM NaCl, 6 mM KCl, 1 mM MgSO_4_, 1 mM Na_2_HPO_4_, 5 mM NaHCO_3_, 5.5 mM glucose, 1 mM CaCl_2_, and 10 mM HEPES, pH 7.4). All reagents were obtained from Sigma-Aldrich, St. Louis, MO, USA.

The neutrophil suspension is suitable for CL measurements only on the day of its isolation. Therefore, the variety of control and experimental conditions used in this study required repeated isolation of fresh neutrophils for each experiment (i.e., different mice were used on different days).

The animal research followed the Guidelines for Ethical Conduct in Animal Care and Use and received approval from the Institutional Animal Care and Use Committee at the Institute of Cell Biophysics under protocol number 57.30.12.2011.

#### 4.2.3. Effect of Changes in the Magnetic Field Parameters on the Distant Interaction During Joint Incubation of Donor and Acceptor

**Figure 7:**
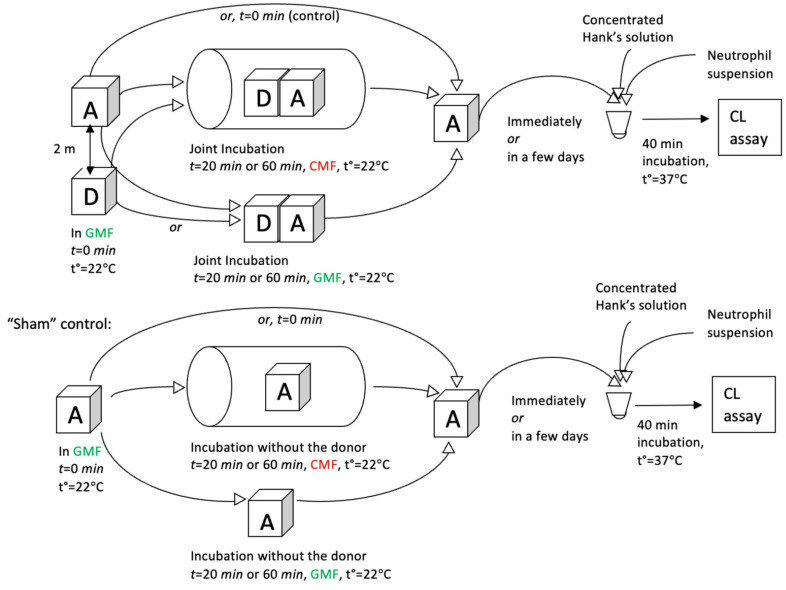
Schematic overview of experimental design to study the effect of changes in the magnetic field parameters on the distant interaction during joint incubation of donor and acceptor. A—acceptor. D—donor. GMF (green)—geomagnetic field; CMF (red)—combined magnetic field.

To assess the effect of the donor on the acceptor, a sample of HD PMA (donor) was incubated at room temperature (23–24 °C) in the presence of an acceptor sample (0.5% ethanol). The plan of the experiment is summarized in Figure 7. Eighteen ml of each sample (donor or acceptor) was placed in identical optical glass cuvettes (Hellma Analytics, Müllheim, Germany, cat. No. 704-001-30-10) with a cap, and jointly incubated in the CMF-generating device with the settings described below. The duration of joint incubation was 20 or 60 min. There were 2 types of controls. As a “sham” control, the acceptor was also incubated for 20 or 60 min in a CMF-generating device without the donor. As another control, a “0 min” incubation of the acceptor without the donor in GMF (outside the CMF-generating device) was used. When donors and acceptors were not jointly incubated, they were kept in the same room at a distance of 200 cm. Using each setting, 6 acceptor samples were prepared, and a 200 µL aliquot of each acceptor was taken for analysis using a cellular chemiluminescence assay (see “Neutrophil suspension preparation for chemiluminescence assay”).

Depending on the task, the samples were incubated in the controlled magnetic field-generating device under one of the following conditions:

1. CMF (combination of SMF at 60 µT and AMF at 100 nT, which is formed according to the sinusoidal regime with a frequency of 48.5 Hz);
2. CMF (combination of SMF at 60 µT and AMF at 100 nT, which is formed according to the sinusoidal regime with a frequency of 12.6 Hz);
3. GMF (SMF of Earth is slightly less than 45 µT, AMF of 50 nT with a frequency of 50 Hz).

In all these set-ups, the 2 types of the abovementioned control measurements were carried out.

#### 4.2.4. Study of Relaxation of HD PMA Donor

**Figure 8:**
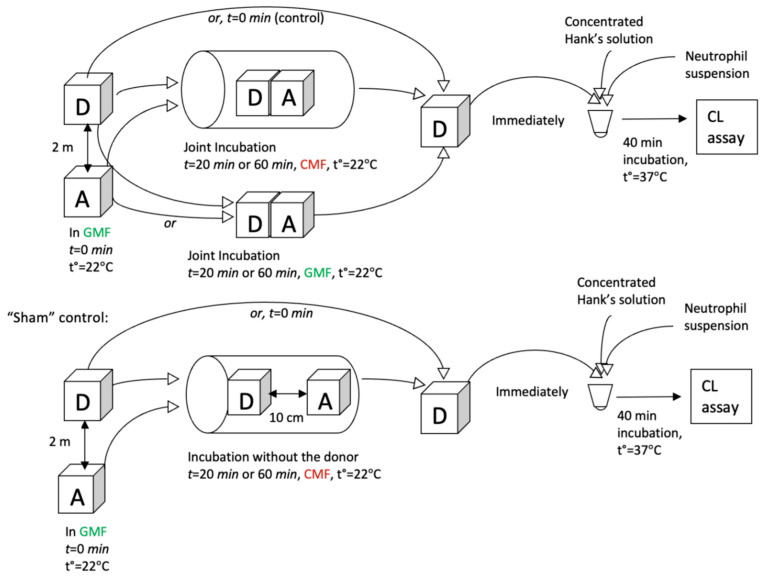
Schematic overview of experimental design to study the relaxation of HD PMA donor. A—acceptor, D—donor. GMF (green)—geomagnetic field; CMF (red)—combined magnetic field.

The ability of a donor (HD PMA) to activate an acceptor (0.5% ethanol) after the joint incubation was estimated on the day of preparation and 6 and 7 days afterward. The design of the experiment is presented in Figure 8.

The HD PMA donor was incubated for 20 and 60 min with the acceptor sample under CMF (a combination of SMF (60 μT) and AMF (100 nT), which is formed according to the sinusoidal regime with a frequency of 12.6 Hz). In the control, the acceptor (“sham” control) was incubated for 20 or 60 min, but without the donor. The second control implied a “0 min” incubation of the acceptor without the donor in the GMF (outside the controlled magnetic field-generating device). Using each setting, 6 acceptor samples were prepared, and a 200 µL aliquot of each acceptor was taken for analysis by cellular chemiluminescence assay (see “Neutrophil suspension preparation for chemiluminescence assay”). In all experiments, an acceptor was freshly prepared.

In another series of experiments, the continuation of the ability of the donor (HD PMA) to act as a respiratory burst activator itself after it was incubated with the acceptor was studied. CMF with SMF at 60 µT and AMF at 100 nT, which is formed according to the sinusoidal regime at a frequency of 12.6 Hz, was chosen for this experiment.

An aliquot of HD PMA solution that had already been used for joint incubation for 20 or 60 min was added directly to the neutrophil suspension. The “control” sample was 0.5% aqueous ethanol solution that did not interact with the donor sample (i.e., “0 min” incubation time, under GMF conditions). Here, in a “sham control” test, the donor was incubated with the acceptor in the CMF (“0 min” values) at a distance of 10 cm from each other. The donor and acceptor samples were prepared and jointly incubated the same day they were prepared and immediately used in the experiment.

Using each setting, 6 acceptor samples were prepared, and a 200 µL aliquot of each acceptor was taken for analysis by cellular chemiluminescence assay (see “Neutrophil suspension preparation for chemiluminescence assay”). It should also be noted that all experiments were carried out in a blind manner, in which samples of 0.5% ethyl alcohol solution in water were prepared and coded by one person and the incubation of neutrophils with these samples and further CL analysis were carried out by other experimenters.

#### 4.2.5. Neutrophil Suspension Preparation for Chemiluminescence Assay

Neutrophil suspension was incubated with samples of specially prepared 0.5% aqueous ethanol solution to assess the ability of these samples to pre-activate or deactivate neutrophils.

To prepare the mixture of neutrophil suspension with samples, three components were mixed: a water-based experimental acceptor sample (200 µL), 5× concentrated Hank’s solution (50 µL), and neutrophil suspension in Hank’s medium (50 µL).

The addition of concentrated Hank’s solution (50 µL) to the acceptor 0.5% aqueous ethanol sample (200 µL) allowed us to create isotonic conditions. Next, a suspension of neutrophils (300,000 cells in 50 μL), which was previously prepared in Hank’s medium, was added to the prepared isotonic mixture. Then the total volume (300 µL) was transferred directly into a 5 mL CL measurement cuvette (plastic round-bottom tubes sized 55 by 12 mm, catalog number 55.484, Sarstedt, Germany) and incubated at 37 ± 0.1 °C for 40 min. Then the CL assay was carried out. The temperature was controlled using a UH4 circulation bath (MLW, Germany).

### 4.3. Chemiluminescent Assay

After the incubation of the mixture of neutrophil suspension with the samples, as described above, the cuvette was placed into the chemiluminometer, and measurement was started. After 60 s of background recording, a solution of luminol (Enzo Life Sciences, Farmingdale, NY, USA) at a final concentration of 0.35 mM, as well as an inducer of ROS generation by neutrophils and a chemotactic formylated peptide N-formyl-Met-Leu-Phe (fMLF) (Sigma, USA) at a final concentration of 2 µM, were added to cuvettes. Measurements were carried out at a temperature of 37 °C and a pH of 7.4. A Lum-1200 chemiluminometer (DISoft LLC, Moscow, Russia) was used for measurements. PowerGraph software (version. 3.3.9, DISoft LLC, Russia) was used for processing the results. The intensity values at the maximum of the CL kinetics curve were recorded. Data are expressed as a percentage of “0-min incubation” controls that were set to 100%. This is CL in the cuvettes to which the acceptors that were not jointly incubated with the donor and were kept under GMF conditions were added. Such normalization is necessary for an adequate comparison of the results of experiments performed on neutrophils obtained from different animals.

### 4.4. Statistical Analysis

The analysis and visualization of the obtained data were carried out using the statistical computing environment R (version 4.0.2., R Foundation for Statistical Computing, Vienna, Austria) and MS Office Excel (version 2410, build 16.0.18129.20158). Data are presented as descriptive statistics and are reported as the arithmetic mean ± standard deviations (SD). The normality of distribution was assessed using the Shapiro–Wilk test, and the homogeneity of variances was assessed using the Bartlett test. A comparison of the groups was carried out using Student’s *t*-test (Welch’s *t*-test in the absence of homogeneity of variances). *p*-values were calculated unadjusted and with Holm’s adjustment for multiple comparisons. Differences were considered statistically significant at *p* < 0.05.

## 5. Conclusions

1. HD PMA prepared using a method of serial dilutions, with manual mechanical shaking between each dilution, acts as a donor for another sample (0.5% aqueous ethanol), which is an acceptor, when they are jointly incubated at a distance of 0 cm under the conditions of CMF or GMF. An aliquot of the acceptor, when added to the neutrophil suspension, causes an increase in luminol-enhanced CL. Acceptors that were incubated for 60 min with the donor caused a significantly higher increase in CL than those that were incubated for 20 min.
2. There was no CL increase in the case in which the donor and the acceptor were kept at a distance of 10 cm in CMF or GMF, nor was there an increase after 20 or 60 min of incubation.
3. The ability of the donor to activate the acceptor was shown to be limited to 6 days after the preparation of the donor.
4. After the donor has been jointly incubated with the acceptor, the donor has a markedly reduced ability to activate neutrophils. The longer incubation leads to a stronger reduction.

## Data Availability

All data supporting the reported results of this study are available from the corresponding author upon reasonable request.

## References

[1] Gonet B. (1985). Influence of constant magnetic fields on certain physiochemical properties of water. Bioelectromagnetics.

[2] Lednev V.V. (1991). Possible mechanism for the influence of weak magnetic fields on biological systems. Bioelectromagnetics.

[3] Tai C.Y., Wu C.-K., Chang M.-C. (2008). Effects of Magnetic Field on the Crystallization of CaCO_3_ Using Permanent Magnets. Chem. Eng. Sci..

[4] Szcześ A., Chibowski E., Hołysz L., Rafalski P. (2011). Effects of static magnetic field on water at kinetic condition. Chem. Eng. Process. Process Intensif..

[5] Chen B., Ivanov I., Klein M.L., Parrinello M. (2003). Hydrogen Bonding in Water. Phys. Rev. Lett..

[6] Cai R., Yang H., He J., Zhu W. (2009). The Effects of Magnetic Fields on Water Molecular Hydrogen Bonds. J. Mol. Struct..

[7] Yan Z., Kong X.Y., Zhu Z., Xiao H. (2024). Magnetic field modulation of confined water structure and transport in nanochannel. J. Mol. Liq..

[8] Semikhina L.P., Kiselev V.F. (1988). Effect of Weak Magnetic Fields on the Properties of Water and Ice. J. Phys. Chem. Solids.

[9] Creanga D.E., Morariu V.V., Isac R.M. (2002). Life in Zero Magnetic Field. IV. Investigation of Developmental Effects on Fruitfly Vision. Electromagn. Biol. Med..

[10] Novikov V.V., Novikov G.V., Fesenko E.E. (2009). Effect of Weak Combined Static and Extremely Low-Frequency Alternating Magnetic Fields on Tumor Growth in Mice Inoculated with the Ehrlich Ascites Carcinoma. Bioelectromagnetics.

[11] Bobkova N.V., Novikov V.V., Medvinskaya N.I., Aleksandrova I.Y., Nesterova I.V., Fesenko E.E. (2018). Effect of Weak Combined Static and Extremely Low-Frequency Alternating Magnetic Fields on Spatial Memory and Brain Amyloid-β in Two Animal Models of Alzheimer’s Disease. Electromagn. Biol. Med..

[12] Fesenko E.E., Yablokova E.V., Novikov V.V. (2024). Weak Magnetic Fields Regulate the Ability of High Dilutions of Water to Enhance ROS Production by Neutrophils. Appl. Sci..

[13] Novikov V.V., Yablokova E.V. (2022). Interaction between Highly Diluted Samples, Protein Solutions and Water in a Controlled Magnetic Field. Appl. Sci..

[14] Manoharan R.R., Zachová K., Buzáš M., Pospíšil P., Křupka M., Prasad A. (2024). NADPH oxidase-dependent free radical generation and protein adduct formation in neutrophils. RSC Adv..

[15] Rossi F., Bellavite P., Berton G., Dri P., Zabucchi G., Basford R. (1982). The Respiratory Burst of Phagocytic Cells: Facts and Problems. Biochemistry and Function of Phagocytes.

[16] Novikov V.V., Yablokova E.V., Fesenko E.E. (2020). The Role of Water in the Effect of Weak Combined Magnetic Fields on Production of Reactive Oxygen Species (ROS) by Neutrophils. Appl. Sci..

[17] Penkov N. (2021). Antibodies processed using high dilution technology distantly change structural properties of IFNγ aqueous solution. Pharmaceutics.

[18] Penkov N., Penkova N. (2020). Analysis of Emission Infrared Spectra of Protein Solutions in Low Concentrations. Front. Phys..

[19] Jerman I., Ogrizek L., Periček Krapež V., Jan L. (2023). Physicochemical Study of the Molecular Signal Transfer of Ultra-High Diluted Antibodies to Interferon-Gamma. Int. J. Mol. Sci..

[20] Jerman I., Ružič R., Krašovec R., Škarja M., Mogilnicki L. (2005). Electrical Transfer of Molecule Information into Water, Its Storage, and Bioeffects on Plants and Bacteria. Electromagn. Biol. Med..

[21] Gorovoy Y.M., Penkov N.V. Distant interaction of supramolecular systems of aqueous solutions as a process of transmission and reception of information and symmetry broadcast. Proceedings of the Conference “Physics of Aqueous Solutions”.

[22] Montagnier L., Del Giudice E., Aïssa J., Lavallee C., Motschwiller S., Capolupo A., Polcari A., Romano P., Tedeschi A., Vitiello G. (2015). Transduction of DNA information through water and electromagnetic waves. Electromagn. Biol. Med..

[23] Ruzic R., Jerman I., Skarja M., Leskovar R., Mogilnicki L. (2008). Electromagnetic Transference of Molecular Information in Garden Cress Germination. Int. J. High Dilution Res..

[24] Foletti A., Ledda M., Lolli M.G., Grimaldi S., Lisi A. (2017). Electromagnetic Information Transfer through Aqueous System. Electromagn. Biol. Med..

[25] Montagnier L., Aissa J., Giudice E.D., Lavallee C., Tedeschi A., Vitiello G. (2011). DNA Waves and Water. J. Phys. Conf. Ser..

[26] Petrova A., Tarasov S., Gorbunov E., Stepanov G., Fartushnaya O., Zubkov E., Molodtsova I., Boriskin V., Zatykina A., Smirnov A. (2024). Phenomenon of Post-Vibration Interactions. Symmetry.

[27] Arani R., Bono I., Giudice E.D., Preparata G. (1995). QED coherence and the thermodynamics of water. Int. J. Modern Phys. B.

[28] Del Giudice E., Voeikov V., Tedeschi A., Vitiello G., Fels D., Cifra M., Scholkmann F. (2015). The origin and special role of coherent water in living systems. Fields of the Cell.

[29] Voeikov V.L., Del Giudice E. (2009). Water respiration-the basis of the living state. Water.

[30] Giudice E.D., Tedeschi A. (2009). Water and autocatalysis in living matter. Electromagn. Biol. Med..

[31] Sen S., Gupta K.S., Coey J.M.D. (2015). Mesoscopic Structure Formation in Condensed Matter Due to Vacuum Fluctuations. Phys. Rev. B.

[32] Giudice E.D., Spinetti P.R., Tedeschi A. (2010). Water dynamics at the root of metamorphosis in living organisms. Water.

[33] Yinnon T. (2020). Liquids Prepared by Serially Diluting and Vigorously Shaking of Aqueous Solutions: Unveiling Effects of the Solute on Their Properties. Water.

[34] Del Giudice E., Tedeschi A., Vitiello G., Voeikov V. (2013). Coherent structures in liquid water close to hydrophilic surfaces. J. Phys. Conf. Ser..

[35] Yinnon T.A., Liu Z.Q. (2015). Domains Formation Mediated by Electromagnetic Fields in Very Dilute Aqueous Solutions: 2. Quantum Electrodynamic Analyses of Experimental Data on Strong Electrolyte Solutions. Water.

[36] Yinnon T.A., Liu Z.Q. (2015). Domains Formation Mediated by Electromagnetic Fields in Very Dilute Aqueous Solutions: 3. Quantum Electrodynamic Analyses of Experimental Data on Solutions of Weak Electrolytes and Non-Electrolytes. Water.

[37] Fang Z., Wang X., Zhou L., Zhang L., Hu J. (2020). Formation and Stability of Bulk Nanobubbles by Vibration. Langmuir.

[38] Liu S., Kawagoe Y., Makino Y., Oshita S. (2013). Effects of Nanobubbles on the Physicochemical Properties of Water: The Basis for Peculiar Properties of Water Containing Nanobubbles. Chem. Eng. Sci..

[39] Gudkov S.V., Penkov N.V., Baimler I.V., Lyakhov G.A., Pustovoy V.I., Simakin A.V., Sarimov R.M., Scherbakov I.A. (2020). Effect of Mechanical Shaking on the Physicochemical Properties of Aqueous Solutions. Int. J. Mol. Sci..

[40] Bunkin N.F., Shkirin A.V., Ninham B.W., Chirikov S.N., Chaikov L.L., Penkov N.V., Kozlov V.A., Gudkov S.V. (2020). Shaking-Induced Aggregation and Flotation in Immunoglobulin Dispersions: Differences Between Water and Water–Ethanol Mixtures. ACS Omega.

[41] Liboff A.R. (2018). The ion cyclotron resonance hypothesis. Bioengineering and Biophysical Aspects of Electromagnetic Fields.

[42] Liboff A.R. (2005). The charge-to-mass ICR signature in weak ELF bioelectromagnetic effects. Advances in Electromagnetic Fields in Living Systems.

[43] Foletti A., Grimaldi S., Lisi A., Ledda M., Liboff A.R. (2013). Bioelectromagnetic medicine: The role of resonance signaling. Electromagn. Biol. Med..

[44] Van der Hart W.J., Van de Guchte W.J. (1988). Excitation of the z-motion of ions in a cubic icr cell. Int. J. Mass Spec. Ion Proc..

[45] Zhadin M., Giuliani L. (2006). Effect of Combined Static and Alternating Magnetic Fields on Ion Motion in a Solution: Theoretical Consideration and Experimental Results. Electromagn. Biol. Med..

[46] Giuliani L., D’Emilia E., Grimaldi S., Lisi A., Bobkova N., Zhadin M.N. (2009). Investigating the ICR Effect in a Zhadin’s Cell. Int. J. Biomed. Sci IJBS.

[47] D’Emilia E., Giuliani L., Lisi A., Ledda M., Grimaldi S., Montagnier L., Liboff A.R. (2014). Lorentz Force in Water: Evidence that Hydronium Cyclotron Resonance Enhances Polymorphism. Electromagn. Biol. Med..

[48] Liboff A.R., Stavroulakis P. (2003). 2.4 Ion Cyclotron Resonance in Biological Systems: Experimental Evidence. Biological Effects of Electromagnetic Fields: Mechanisms, Modeling, Biological Effects, Therapeutic Effects, International Standards, Exposure Criteria.

[49] Liboff A.R. (2019). ION cyclotron resonance: Geomagnetic strategy for living systems?. Electromagn. Biol. Med..

